# MiRNA10b-directed nanotherapy effectively targets brain metastases from breast cancer

**DOI:** 10.1038/s41598-021-82528-2

**Published:** 2021-02-02

**Authors:** Byunghee Yoo, Alana Ross, Pamela Pantazopoulos, Zdravka Medarova

**Affiliations:** MGH/MIT/HMS Athinoula A. Martinos Center for Biomedical Imaging, Department of Radiology, Massachusetts General Hospital and Harvard Medical School, Boston, MA 02129 USA

**Keywords:** Cancer therapy, Metastasis

## Abstract

RNA interference represents one of the most appealing therapeutic modalities for cancer because of its potency, versatility, and modularity. Because the mechanism is catalytic and affects the expression of disease-causing antigens at the post-transcriptional level, only small amounts of therapeutic need to be delivered to the target in order to exert a robust therapeutic effect. RNA interference is also advantageous over other treatment modalities, such as monoclonal antibodies or small molecules, because it has a much broader array of druggable targets. Finally, the complementarity of the genetic code gives us the opportunity to design RNAi therapeutics using computational, rational approaches. Previously, we developed and tested an RNAi-targeted therapeutic, termed MN-anti-miR10b, which was designed to inhibit the critical driver of metastasis and metastatic colonization, miRNA-10b. We showed in animal models of metastatic breast cancer that MN-anti-miR10b accumulated into tumors and metastases in the lymph nodes, lungs, and bone, following simple intravenous injection. We also found that treatment incorporating MN-anti-miR10b was effective at inhibiting the emergence of metastases and could regress already established metastases in the lymph nodes, lungs, and bone. In the present study, we extend the application of MN-anti-miR10b to a model of breast cancer metastatic to the brain. We demonstrate delivery to the metastatic lesions and obtain evidence of a therapeutic effect manifested as inhibition of metastatic progression. This investigation represents an additional step towards translating similar RNAi-targeted therapeutics for the systemic treatment of metastatic disease.

## Introduction

Despite the promise of RNAi therapy, delivery to organs outside the liver remains a challenge. Previously, we developed a therapeutic that could broadly target elements of the cellular RNAi apparatus in tumors and metastases, following simple intravenous injection. These included small interfering RNAs, antagomirs for the suppression of endogenous microRNAs, and microRNA mimics^1–9^.

To shuttle therapeutic oligonucleotides to tumors and metastases, we employed dextran-coated iron oxide nanoparticles optimized in terms of size, charge, and surface coating in order to ensure stability in the circulation, a long circulation half-life, effective accumulation into the interstitium of primary and metastatic tumor lesions through hemodynamic mechanisms, and uptake of therapeutically significant amounts by tumor cells, through endocytosis. While unproven, mechanisms related to “metabolic” targeting of the dextran-coated nanoparticles through the Warburg effect were also hypothesized. The nanoparticles were linked to oligonucleotides by finetuning their surface coating in order to be able to chemically conjugate large amounts of oligo that, while retained onto the nanoparticle surface in the circulation and interstitium, is separated from the nanoparticles in the reducing intracellular environment and is free to enter the RNA induced silencing complex (RISC).

The majority of our work employed an antagomir-based strategy that could effectively inhibit microRNA-10b in primary tumors and metastases from breast cancer^5,8^. MicroRNA-10b is one of the best validated targets in metastasis that has been shown to drive metastatic dissemination^10^ and metastatic cell viability^5^. Multiple clinical studies have proven the value of miR-10b as a biomarker of metastasis in patients with a variety of malignancies, including breast, hepatocellular, lung, pancreatic, colorectal, gastric, ovarian adenocarcinoma, and melanoma, to name a few. MircoRNA-10b has been validated as a biomarker of clinical parameters, such as stage, presence of metastases, relapse-free survival, overall survival, degree of invasion, and time to recurrence (reviewed in^11^).

Our focus on microRNA-10b was motivated not only by its proven clinical relevance as a biomarker of metastatic progression but also by our own discovery that miR-10b is a major driver of metastatic cell viability^3,5,8^. These studies came on the heels of earlier seminal work that established miR-10b as a critical effector of tumor cell migration and invasion and metastatic initiation^10,12^. Combined, the roles of microRNA-10b in metastatic initiation and progression established its importance as a therapeutic target in cancer. For that reason, a major goal of our work has been to develop and test therapeutics based on miR-10b inhibition.

To inhibit miR-10b, we used a therapeutic (termed MN-anti-miR10b), which consisted of LNA-incorporating antagomirs conjugated to dextran-coated iron nanoparticles^13^. We performed thorough mechanistic studies elucidating the biological effects of MN-anti-miR10b^3,5,6^ and showed that MN-anti-miR10b affects molecular processes downstream of E-cadherin and, through its effect on HOXD10 and c-JUN profoundly inhibits the capacity of tumor cells to migrate and invade surrounding tissue and induces apoptosis in metastatic tumor cells. In this earlier work, we tested the effectiveness of the therapeutic in murine models of triple negative breast cancer. We confirmed uptake by primary and metastatic tumor cells, found in the lymph nodes, lungs, and bone, following intravenous injection^8^. When treatment with MN-anti-miR10b was initiated prior to the formation of overt metastases, metastasis was prevented^3^. When treatment was initiated after the formation of detectable metastases, therapy involving MN-anti-miR10b elicited durable regressions with no evidence of systemic toxicity^5,8^. Importantly, after four to six weekly treatments with MN-anti-miR10b in combination with low dose chemotherapy, we observed complete regression of detectable metastatic lesions. At that point, even though therapy was discontinued, metastases did not recur^5,8^.

In our earlier work, we obtained evidence of MN-anti-miR10b uptake by micrometastatic lesions in the brains of mice implanted orthotopically with the highly aggressive 4T1 murine breast cancer cell line^8^. In the present study, we focused more distinctly on the capacity of MN-anti-miR10b to target brain metastatic lesions from breast cancer and to elicit a therapeutic response. We used the MDA-MB-231-Br “brain homing” subclone of the human MDA-MB-231 cell line, which represents a pure clonal population of brain-tropic metastatic cells and therefore permits directed study of therapeutic response in the brain lesions.

## Methods

### Preparation of MN-anti-miR10b

Aminated dextran coated iron oxide nanoparticles (NP) were synthesized using a protocol similar to ones published previously^3,4^. The nanoparticles were labeled with near infra-red dye, Cy5.5, and conjugated to the heterobifunctional linker *N*-succinimidyl 3-[2-pyridyldithio]-propionate (SPDP; Thermoscientific Co.) and activated oligos sequentially. Briefly, Cy5.5-NHS was conjugated to NP to produce Cy5.5-labeled magnetic nanoparticles (MN) overnight. SPDP in anhydrous DMSO was incubated with MN. The anti-miRNA-10b LNA antagomir was modified with the 5′-Thiol-Modifier C6 disulfide (5′-ThioMC6), which was utilized for conjugation to SPDP-modified MN. The disulfide on the oligonucleotide was activated by 3% Tris (2-carboxyethyl) phosphine hydrochloride (TCEP, Thermoscientific Co.), followed by purification with ammonium acetate/ethanol precipitation treatment prior to conjugation to SPDP-modified MN. After TCEP activation and purification, the oligo was dissolved in water and incubated with the SPDP-modified MN overnight. MN-anti–miR-10b was freshly prepared each week.

### Characterization of MN-anti-miR10b

The number of oligos per MN was quantified by gel electrophoresis after the treatment with TCEP. The number of Cy5.5 was determined by UV/VIS spectrophotometry (675 nm, e = 250,000 M^−1^ cm^−1^). The concentration of MN was obtained by iron assay (Pointe Scientific, Canton, MI). The size of MN was measured using transmission electron microscopy (TEM; JEOL 2100, Tokyo, Japan; 200 kV, 0.19 nm resolution) in order to determine the diameter of the iron-oxide core and dynamic light scattering (Malvern Panalytical, Malvern, UK) in order to determine the hydrodynamic (Z-average) diameter, as described previously^3, 4^.

### Cell lines

Human brain-tropic MDA-MB-231-BrM2-831 cells were kindly provided by Dr. Joan Massagué (Memorial Sloan Kettering Cancer Center, Manhattan, NY). The cells are transduced with a lentivirus expressing a triple-fusion reporter encoding herpes simplex virus thymidine kinase 1, green fluorescent protein (GFP) and firefly luciferase (Luc). The cells were cultured in DMEM supplemented with l-glutamine, 10% FBS and antibiotics (1000 units/mL penicillin and 1000 U/mL streptomycin) as recommended by the provider^14^.

### Animal model

Ten female Balb/c nude mice (8 weeks old, The Jackson Laboratory; Bar Harbor, ME) were directly injected into the heart with MDA-MB-231-BrM2-831 cells (0.175 × 10^6^ cells in 100 mL HBSS)^15^. Briefly, after cleaning the skin over the chest, a needle was inserted 2 mm to the left of the sternum and 12 mm above the tip of the xiphoid process. The tip of the needle was slightly in the caudal direction and was angled 25° relative to the sagittal plane^16^. Since the cells express green fluorescent protein (GFP) and luciferase (Luc), they can be detected by noninvasive optical imaging for the quantification of metastatic tumor burden. All animals were scanned weekly in order to monitor tumor growth and metastasis in the brain. Finally, MN-anti-miR-10b was administered after the confirmation of breast cancer metastases in the brain and continued every week following the pre-fixed schedule^5,8^. Animals were sedated under Isoflurane anesthesia 10 min before cardiac cell injection, peritoneal injection or imaging. For post-procedural pain relief, animals were administered buprenorphine twice daily for the duration of the experiment (i.e., approximately 2 weeks post-cardiac inoculation). All procedures were carried out in aseptic fashion in clean facilities. Animals were observed daily for their level of activity and normal eating, drinking and grooming behavior. According to AVMA guidelines, a high dose of sodium pentobarbital was injected intraperitoneally (200 mg/kg IP) to sacrifice animals at the end of study. Animals were housed in the MGH Animal Facility, which is under the supervision of the MGH Office of Laboratory Animal Research. All animal experiments were performed in compliance with institutional guidelines and approved by the Institutional Animal Care and Use Committee at Massachusetts General Hospital (Boston, MA).

### Fluorescence optical imaging (FLI)

Near infra-red fluorescence imaging was performed using the IVIS Spectrum imaging system (Perkin Elmer, Hopkinton, MA). Anesthetized mice were injected i.v. with Cy5.5 labeled MN-anti-miR10b (30 mg/kg as iron, 20 mg/kg as oligo) and scanned by fluorescence reflectance imaging 24 h later. The acquisition conditions are summarized as follows: Exposure time, 0.5 s; Binning factor, 8; Excitation filter range, 675 nm; Emission filter range, 720 nm; f number, 2. Tumor location and growth were monitored by fluorescence reflectance imaging using filters for green fluorescence protein (GFP). The acquisition conditions were: Exposure time 4 s; Binning factor, 8; Excitation filter range, 465 nm; Emission filter range, 540 nm; f number, 2. All images were analyzed using the Living Image Software (ver 4.5, IVIS Spectrum, Perkin Elmer, Hopkinton, MA). Radiant efficiency of the excised tissues was used for signal quantification^17^.

### Bioluminescence optical imaging (BLI)

BLI was used to evaluate tumor burden. Imaging was performed using the IVIS Spectrum imaging system. Anesthetized mice were injected intraperitoneally with d-luciferin potassium salt in DPBS (200 mL of 15 mg/mL; Perkin Elmer, Hopkinton, MA) 12 min before image acquisition. Identical image acquisition settings (time, ~ 0.5–60 s; F-stop, 2; binning, medium) and the same ROI were used to obtain total radiance (photons/s) over the ROI. BLI was performed for about 6–15 min to obtain the maximum radiance. All images were processed using the Living Image Software (ver 4.5, IVIS Spectrum, Perkin Elmer, Hopkinton, MA). Total flux from the bioluminescence readings was used for signal quantification^5^.

### Treatment

Treatment was delivered to animals immediately after the confirmation of breast cancer metastases in the brain by BLI. The treatment protocol consisted of injection of MN-anti-miR-10b or MN nanoparticles without antagomir intravenously (30 mg/kg as iron, 20 mg/kg as oligo) every week. Treatment was administered weekly for 3 weeks and was stopped to analyze the distribution of breast cancer metastases and the uptake of MN-anti–miR-10b in breast cancer metastases in the brain.

### Microscopy

Excised tissues were embedded in Tissue-Tek OCT compound (Sakura Finetek, Torrance, CA) and snap frozen in liquid nitrogen. The tissues were cut into 7 µm sections and fixed in 4% formaldehyde for 10 min. The slides were analyzed using a Nikon E400 fluorescence microscope (Nikon, Tokyo, Japan), equipped with the necessary filter sets (MVI Inc., Avon, MA). Images were acquired using a charge coupled device camera with near-IR sensitivity. The images were analyzed using ImageJ (Ver. 1.51c, NIH).

For the HOXD10 staining, tissues were incubated with recombinant anti-HOXD10 antibody [EPR9374] (ab138508), followed by fluorescently-labeled anti-rabbit secondary polyclonal antiserum (Abcam, Cambridge, MA). The slides were analyzed by fluorescence microscopy as described above.

### Statistical analysis

Data were expressed as mean ± SD. Statistical comparisons were made using a two-tailed t-test (SigmaStat 3.0; Systat Software, Richmond, CA). A value of *p* < 0.05 was considered statistically significant.

## Results

### Characterization of MN-anti-miR10b

**Figure 1:**
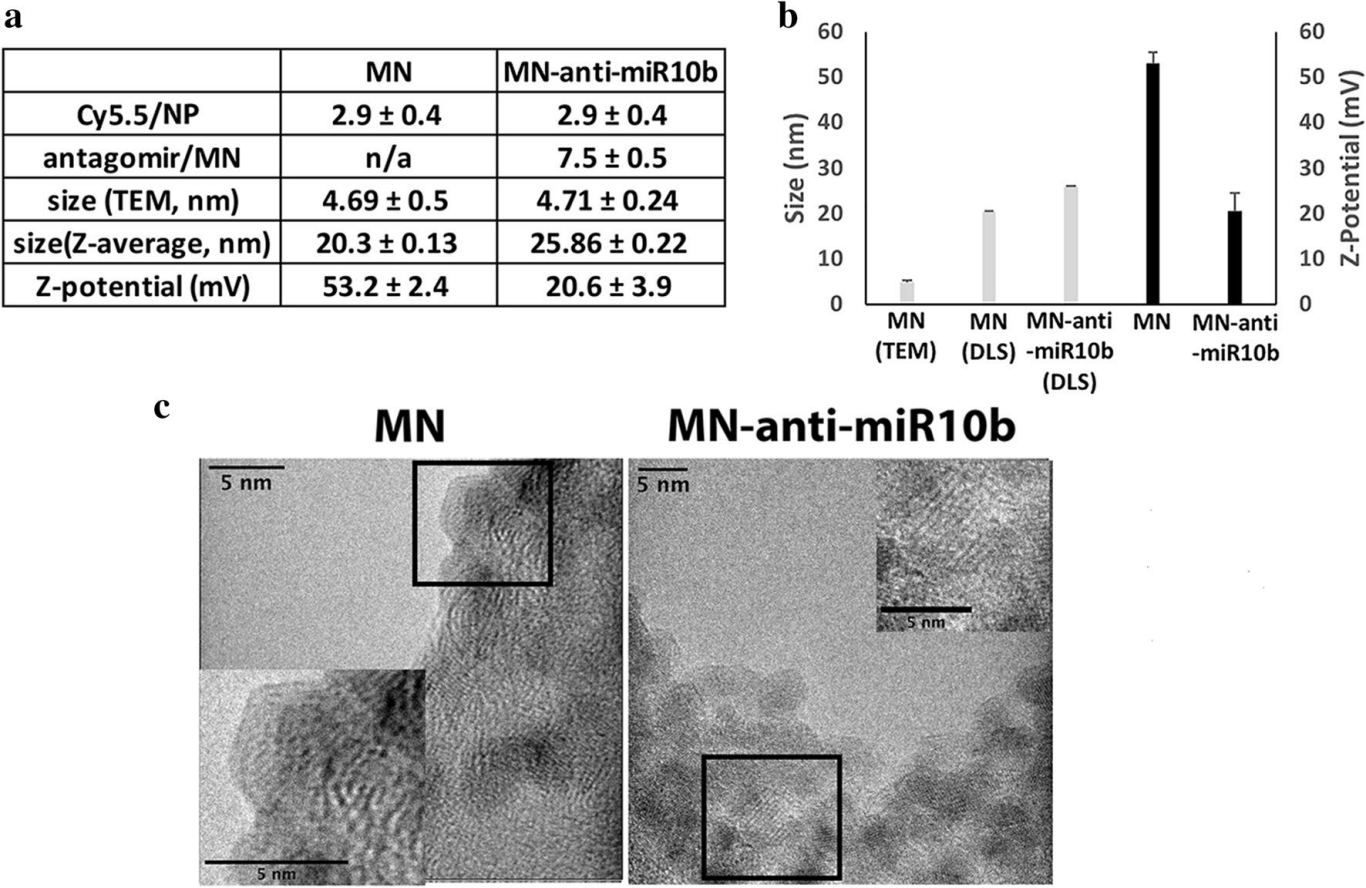
Synthesis and characterization of MN-anti-miR10b. (**a**) Characterization of the nanotherapeutic, which consisted of aminated dextran-coated iron oxide nanoparticles covalently conjugated to the Cy5.5 fluorescent dye and antagomirs against miR-10b. (**b**) Dynamic light scattering (DLS) to measure hydrodynamic diameter and zeta potential. The hydrodynamic diameter of MN-anti-miR10b was larger than that of the MN precursor. The zeta potential of MN-anti-miR10b was less positive than that of the oligo-deficient MN precursor (Unpaired t-test; n = 3; *p* ≤ 0.05). (**c**) Transmission electron microscopy (TEM) to measure iron core size and visualize lattice structure. Both the MN-anti-miR10b nanotherapeutic and the precursor MN nanoparticles had equivalent iron core sizes and similar lattice structures.

In this study, we derivatized dextran-coated magnetic nanoparticles (NP) to introduce amino groups onto the surface of the nanoparticles and functionalize them with a near infrared fluorescent dye (Cy5.5) to generate the magnetic nanoparticle template, MN. Anti-miR10b antagomirs were conjugated to MN to produce MN-anti-miR10b, using a protocol similar to ones previously described^4,13^. The number of amino groups per NP was determined as 74. A near infra-red fluorescent dye, Cy5.5 was loaded onto NP, to generate MN. The ratio of Cy5.5 per MN was found to be 2.9 ± 0.4. Following conjugation to the anti-miR10b antagomir to generate MN-anti-miR10b, the number of antagomirs per MN was determined as 7.5 ± 0.5 (Fig. 1a). The size and the zeta potential of MN were 20.3 ± 0.13 nm and 53.2 ± 2.4 mV, respectively (Fig. 1a,b). The hydrodynamic (Z-average) diameter and the zeta potential of MN-anti-miR10b were determined as 25.86 ± 0.22 nm and 20.6 ± 3.9 mV, respectively (Fig. 1a,b). The hydrodynamic diameter of MN-anti-miR10b was increased by 5.5 nm after functionalization. The increased hydrodynamic diameter and the reduced zeta potential, following conjugation to the antagomir, implied that the antagomir was located on the outer surface of MN. The MN-anti-miR10b nanotherapeutic was highly monodisperse with a polydispersity index (PDI) of 0.17. Considering the diameter of the iron oxide core (4.69 ± 0.5 nm, Fig. 1c), the amine-derivatized dextran coat formed an outer shell with a thickness of 7.8 nm. Finally, TEM confirmed that the lattice structure of the nanoparticles was preserved following functionalization (Fig. 1c).

### Delivery of MN-anti-miR10b to brain metastases

**Figure 2:**
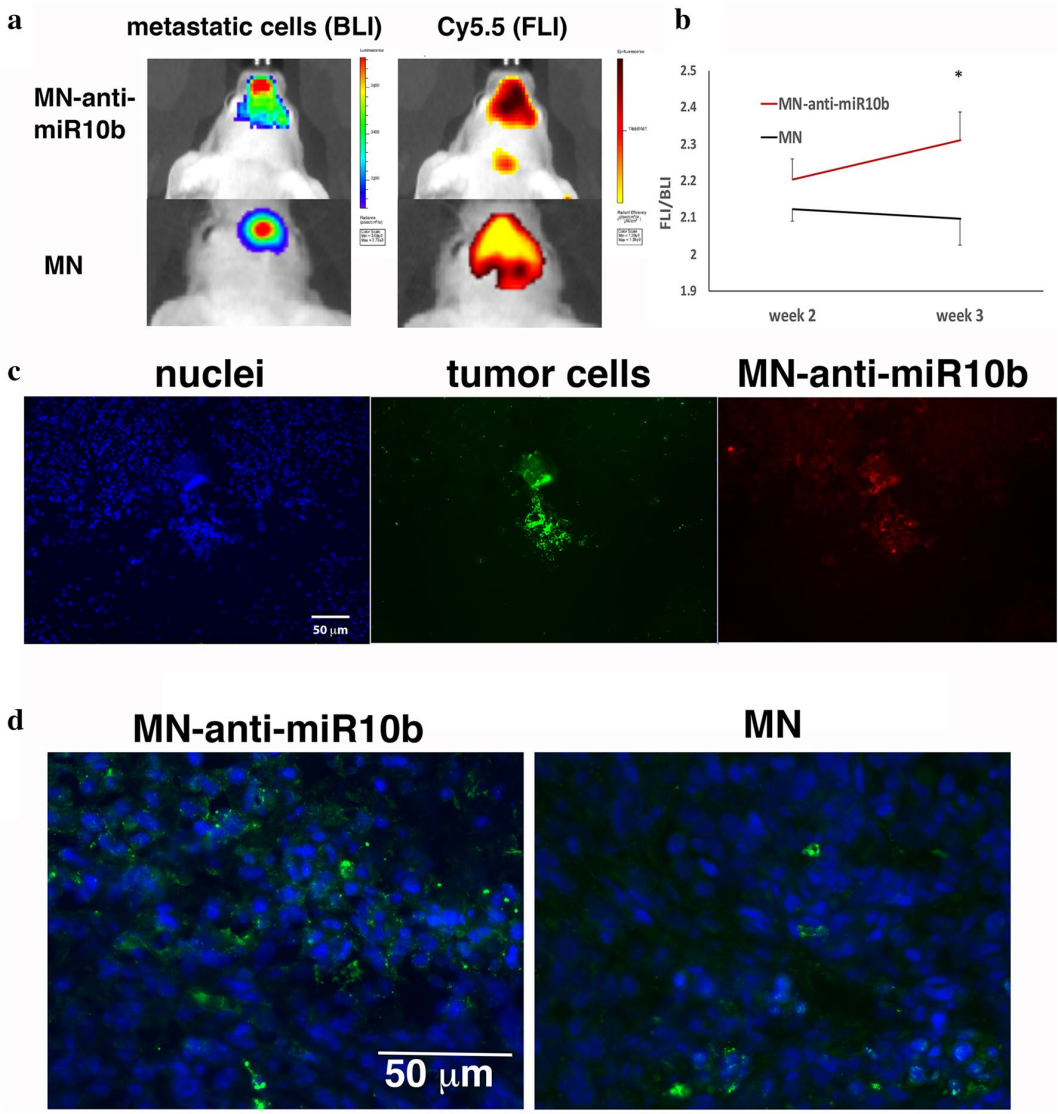
Delivery of MN-anti-miR10b to brain metastases from breast cancer. (**a**) In vivo bioluminescence (metastatic cells) and Cy5.5 fluorescence (MN-anti-miR10b) images over the brain of a mouse treated with MN-anti-miR10b obtained 24 h. after injection of the nanotherapeutic. Bioluminescence and Cy5.5 fluorescence co-localized indicating selective accumulation of MN-anti-miR10b in the metastatic lesions. (**b**) Ratio of fluorescence intensity (FLI: Relative Fluorescence Units, MN-anti-miR10b) to bioluminescence (BLI: photons/sec, metastatic burden) indicating that MN-anti-miR10b accumulation increased in the brain lesions with treatment, which was not observed in the MN-treated controls. (**c**) Fluorescence microscopy of brain metastatic lesions demonstrating the presence of micrometastases in perivascular regions of the brain and the uptake of MN-anti-miR10b by the micrometastases. (**d**) Fluorescence microscopy of brain metastatic lesions demonstrating upregulation of Homeobox D10 (HoxD10) with treatment and confirming target engagement by the MN-anti-miR10b therapeutic (Pearson correlation; *n* = 5; *p* ≤  0.05).

The MDA-MB-231-BrM2-831 breast cancer cell line is transduced with a lentivirus expressing a triple-fusion reporter. The reporter encodes herpes simplex virus thymidine kinase 1 suitable for detection by nuclear imaging, green fluorescent protein (GFP) optimal for detection by microscopy, and firefly luciferase (Luc) optimal for detection by bioluminescence optical imaging (BLI)^15^. To generate metastases to the brain, MDA-MB-231-BrM2-831 cells were administered by direct cardiac injection. One week after cell injection, all mice were monitored in order to confirm the formation of metastases. Brain metastases were clearly visible by BLI 1 week after cell injection. At that point, MN-anti-miR10b was injected intravenously. Twenty four hours later, the animals were imaged by noninvasive bioluminescence and fluorescence optical imaging with filters appropriate for Cy5.5. As seen in Fig. 2a, the MN-anti-miR10b co-localized with the metastatic lesions, indicating lesion-selective accumulation in the brain (Fig. 2a). The ratio of fluorescence intensity (MN-anti-miR10b) to bioluminescence flux (tumor burden) increased in week 3 compared to the earlier time point and the ratio was significantly higher than in MN-treated controls (Fig. 2b). This result indicated that a higher amount of MN-anti-miR10b was entrapped in the brain metastases in the course of treatment, consistent with a reduced rate of dilution of the therapeutic and reflecting slower tumor cell proliferation.

Accumulation of MN-anti-miR10b in the brain metastatic lesions was confirmed by fluorescence microscopy. We observed perivascular micrometastatic lesions from the GFP-expressing cell line, which also accumulated MN-anti-miR10b (Fig. 2c).

To establish target engagement, we analyzed the expression of Homeobox D10 (HOXD10) by immunofluorescence. HOXD10 is a known miR-10b target, which demonstrates an inverse correlation with miR-10b expression^10,12^ and is commonly used to demonstrate target engagement, following treatment with miR-10b inhibitors. Consistent with the known mechanism of action of miR-10b, inhibition of the target by MN-anti-miR10b upregulated HOXD10 in brain metastases (Fig. 2d).

### Therapeutic effect of MN-anti-miR10b on brain metastases from breast cancer

**Figure 3:**
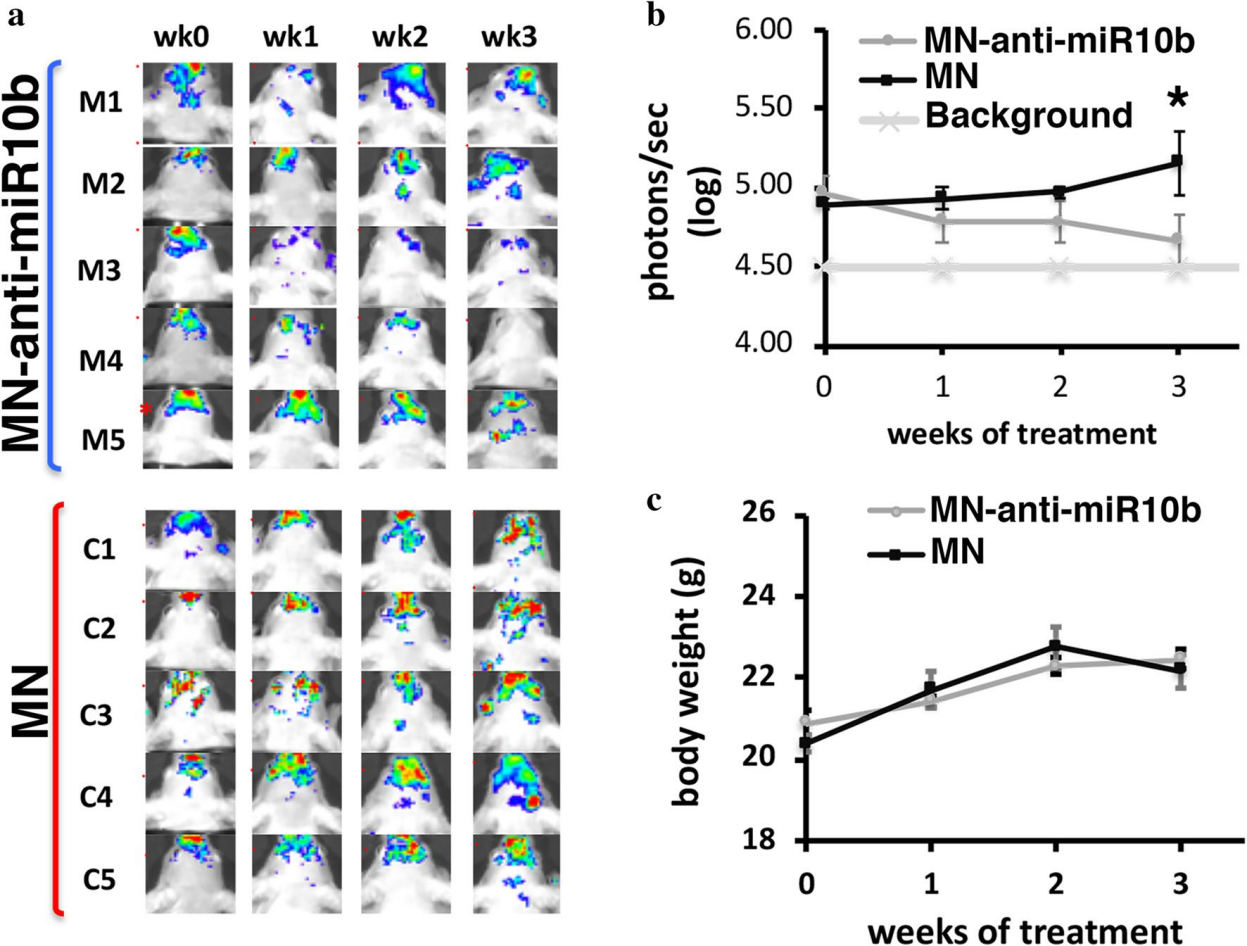
Therapeutic effect of MN-anti-miR10b in a murine model of brain metastatic breast cancer. (**a**) Bioluminescence images of mice treated with MN-anti-miR10b showing clear regression of the lesions in the course of treatment with the nanotherapeutic. By contrast, the metastatic lesions in the MN-treated controls visibly progressed over the course of the study. M1–M5: experimental animals; C1–C5: control animals. (**b**) Bioluminescence radiance demonstrating significantly lower metastatic burden in the MN-anti-miR10b-treated versus the MN-treated control animals by 3 weeks after the beginning of therapy and a tendency towards metastatic regression with treatment in the experimental animals. (**c**) Body weight of the experimental and control animals during treatment showing lack of evidence of morbidity associated with the treatment (t-test; n = 5; *p = 0.047; ns – *p* > 0.05).

Using a treatment protocol established by us previously^5^, the animals with BLI-detectable brain metastases were injected weekly with MN-anti-miR10b, applied as monotherapy. Treatment was continued for an average of 3 weeks, at which point outgrowth of the metastases in the control animals necessitated termination of experiments. Metastatic burden was measured by bioluminescence imaging. Reduction in metastatic burden in the brain was detectable even after the first treatment (Fig. 3a). After 3 weeks of treatment, metastatic burden was significantly lower in the MN-anti-miR10b-treated group versus the MN-treated control group (Fig. 3a,b). In the experimental animals treated with MN-anti-miR10b, metastatic burden at the end of treatment was significantly lower than at the initiation of treatment, indicating that viable lesion mass regressed during treatment (Fig. 3b). Finally, body weight was not different between experimental and control mice (Fig. 3c) confirming our prior conclusions that the nanotherapeutic is not associated with morbidity at the injected dose^5^.

## Discussion

The present manuscript describes new findings in a series of studies relevant to microRNA-10b as a critical therapeutic target in metastatic disease. Previously, we designed and tested the miR-10b targeted nanotherapeutic MN-anti-miR10b in animal models of lymph node metastatic breast cancer and advanced metastatic breast cancer^5,8^. We also demonstrated in a panel of human cancer cell lines that MN-anti-miR10b is effective across cancer types^6^.

Here, we apply MN-anti-miR10b in a model of human breast cancer metastatic to the brain. The importance of the work is twofold. First, it demonstrates successful delivery of the therapeutic to metastatic lesions in the brain. This suggests that the blood–brain barrier is sufficiently disrupted to allow passage of MN-anti-miR10b across the vascular endothelium of the metastatic lesions and permit access to the tumor cells. Second, the present study shows that the biological significance of miR-10b as a driver of metastatic progression is preserved in brain-tropic metastatic tumor cells.

The present study represents a novel advancement in the context of our prior work because delivery to brain metastatic lesions presents a distinct challenge due to the partially disrupted blood–brain barrier. Nanoparticle-assisted drug delivery across the BBB has been demonstrated in the past. For example, lipid nanoparticles have proven successful because of uptake through lipid-mediated free diffusion^18^. Polymeric nanoparticles are transported across the BBB via receptor-mediated endocytosis^19^. However, unmodified iron oxide nanoparticles do not readily cross the BBB. In an in vitro BBB model system, unmodified 15-nm iron oxide nanoparticles were taken up by the intact BBB with an efficiency of only 30%^20^. While the model that we are studying here is not characterized by an intact blood–brain barrier, our findings suggest the feasibility of targeting metastatic lesions in the brain using similar constructs.

A key point that needs to be addressed has to do with the feasibility of using MN-anti-miR10b as monotherapy against breast cancer metastatic to the brain. In our prior studies, at lower doses, MN-anti-miR10b was very effective at preventing the emergence of detectable macrometastatic disease in a model of lymph-node metastatic breast cancer and could induce arrest of metastatic progression^3^. In the same model, combination with low-dose chemotherapy potentiated the effect of the nanotherapeutic and led to actual regression of metastases^5^. This observation was corroborated in a model of advanced metastatic breast cancer with metastases to the lungs and bone^8^. Based on these results, we hypothesized that low-dose chemotherapy synergized with MN-anti-miR10b to inhibit the cell cycle in the metastatic tumor cells and permit the attainment of higher effective concentrations of the MN-anti-miR10b therapeutic in the cells by reducing the rate of cell division. Based on this hypothesis, we posited that the therapeutic could be successful at regressing metastatic disease as monotherapy when delivered at higher concentrations and that the minimal effective dose (MED) is likely proportional to the rate of cell division in the metastatic lesions. The cell line used in the present study and the cell line used in our prior studies on lymph-node metastatic breast cancer are both MDA-MB-231 sublines with comparable growth kinetics. However, the dose of the therapeutic used in the current study as monotherapy was double the dose used in the prior investigation, in which combination with a cytostatic was necessary to induce regression^5^. While this is not a controlled side-by-side comparison, it implies that MN-anti-miR10b could be effective as monotherapy at higher doses, optimized for the growth kinetics of the metastatic lesions.

In summary, we are presenting new evidence of the utility of our miR-10b targeted therapeutic, MN-anti-miR10b, for the treatment of metastatic breast cancer. We further validate the fundamental and unique value of miRNA-10b as a therapeutic target for the tangible regression of established metastases. This, in addition to similar effective and clinically-relevant vehicles, represents a modality for cancer treatment that is radically different from other modern therapies, such as monoclonal antibodies and small molecules, both in terms of the underlying molecular biology and the potential for rational delivery to target organs.
